# Comprehension and Eye Movements in the Processing of Subject- and Object-Relative Clauses: Evidence from Dyslexia and Individual Differences ^†^

**DOI:** 10.3390/brainsci11070915

**Published:** 2021-07-10

**Authors:** Marianna Stella, Paul E. Engelhardt

**Affiliations:** 1 School of Social Sciences and Humanities, University of Suffolk, Ipswich IP4 1QJ, UK; 2 School of Psychology, University of East Anglia, Norwich NR7 7TJ, UK; p.engelhardt@uea.ac.uk

**Keywords:** developmental dyslexia, reading disability, eye movements, sentence processing, sentence comprehension

## Abstract

In this study, we examined eye movements and comprehension in sentences containing a relative clause. To date, few studies have focused on syntactic processing in dyslexia and so one goal of the study is to contribute to this gap in the experimental literature. A second goal is to contribute to theoretical psycholinguistic debate concerning the cause and the location of the processing difficulty associated with object-relative clauses. We compared dyslexic readers (*n* = 50) to a group of non-dyslexic controls (*n* = 50). We also assessed two key individual differences variables (working memory and verbal intelligence), which have been theorised to impact reading times and comprehension of subject- and object-relative clauses. The results showed that dyslexics and controls had similar comprehension accuracy. However, reading times showed participants with dyslexia spent significantly longer reading the sentences compared to controls (i.e., a main effect of dyslexia). In general, sentence type did not interact with dyslexia status. With respect to individual differences and the theoretical debate, we found that processing difficulty between the subject and object relatives was no longer significant when individual differences in working memory were controlled. Thus, our findings support theories, which assume that working memory demands are responsible for the processing difficulty incurred by (1) individuals with dyslexia and (2) object-relative clauses as compared to subject relative clauses.

## 1. Introduction

The purpose of the current study is to investigate the processing of subject- and object-extracted relative clauses, henceforth referred to as subject and object relatives (see Table 1 for examples). Past research has identified that object relatives are consistently more difficult than subject relatives (e.g., [1,2,3]). We are interested in examining how individuals with dyslexia process these kinds of sentences because research into sentence processing in dyslexia is extremely limited, and thus, the first goal of the study is to determine whether individuals with dyslexia have difficulties with this particular type of syntactic construction. The second goal of the study is to contribute to the theoretical debate concerning the source of processing difficulty between subject and object relatives. Theoretical debates have identified two key issues: the first is violation of predictive expectations, which have been computationally assessed via Surprisal [4,5], and is very closely related to linguistic prediction (for reviews see [6,7]). The second source of difficulty is working memory. With object relatives, the object noun phrase must be held in memory until the reader encounters the relative clause verb, with which it is associated [1,3,8,9,10,11,12,13]. Thus, resolving the long-distance dependency is expected to incur substantial demand on cognitive resources, especially in terms of working memory. Dyslexia presents a very interesting test of these theoretical debates, because dyslexia has been associated with deficits in both working memory [14,15] and linguistic prediction [16]. Thus, there is good reason to suspect that individuals with dyslexia will show both online processing and offline comprehension deficits with object-relative sentences.

In the remainder of the Introduction, we first cover the literature on dyslexia with a particular focus on sentence comprehension in dyslexia and what is known about the eye movement behaviour of individuals with dyslexia when they read. We then turn our attention to the theoretical psycholinguistics literature, and the two broad classes of processing models (memory-based and expectation-based) that make predictions about the processing difficulty associated with subject- and object-relative sentences. Finally, we present the rationale and hypotheses of the current study.

### 1.1. Sentence Processing in Dyslexia

Dyslexia is a specific learning disability that has a neurobiological origin and is primarily characterised by deficits in phonological skills. These deficits manifest as difficulties in single-word decoding and spelling, as well as in reading accuracy and fluency issues [17,18]. Phonological skill deficits affect an individual’s ability to manipulate, store and retrieve the phonemic and graphemic codes of language [19]. Studies on dyslexia have reported syntactic issues in both oral and written language across the lifespan [20,21]. Impairments in the comprehension and production of complex syntax may originate from several sources. These range from broad weakness in language processing [22] to more specific linguistic deficits, such as, phonological skills and/or semantics. Other studies have also suggested that deficits in dyslexia may arise from more basic cognitive abilities/executive functions, such as working memory [23,24]. Finally, it is important to keep in mind that many individuals with dyslexia do not read as much as typically developed individuals, and so, deficits in dyslexia may also be a secondary result of reduced reading experience [25].

The current literature on sentence processing in dyslexia is extremely limited. This is important because we do not know whether dyslexic readers show difficulty in sentence processing and sentence comprehension, over and above single-word decoding difficulties [26,27]. There are considerable differences between reading single words and reading sentences. Comprehending sentences requires the ability to combine words together into meaningful phrases and extract compositional meaning, and is therefore, considerably different and more complex than single-word reading.

There have been several studies that have examined the eye movements of individuals with dyslexia, from investigating the basis of Pavlidis’ [28] theory that atypical eye movements are the cause of dyslexia to the association between oculomotor control, visuo-spatial deficits [29,30,31] and differences in saccadic eye movements [32,33]. Further studies on eye movements of individuals with dyslexia reading single words and non-words [34,35], sentences [36,37,38] and texts [26,27] have shown that dyslexic readers tend to make longer fixations, shorter saccades and a greater proportion of regressive eye movements compared to non-dyslexic readers.

As mentioned previously, individuals with dyslexia show deficits in several areas, which fall along a continuum and are assumed to be linked to their problems with reading. In the current study, we focused on two key individual differences variables, which were assessed along with sentence comprehension and eye movements. The first was working memory [14] and the second was verbal intelligence [39,40,41]. We assumed that these two individual difference variables would play a role in the processing and comprehension of sentences with object-relative clauses. In order to read and understand a sentence, people need to be able to store and process information at the same time, as it requires them to combine prior information provided in the sentence to make inferences and resolve long-distance dependencies [42]. Working memory has been suggested as a key factor in the successful comprehension of object-relative clauses [9], and individuals with dyslexia often have deficits in working memory [14,15].

With respect to verbal intelligence, reading requires a broad vocabulary in order to quickly extract the correct meaning of words, and in turn, the meaning of sentences. According to Perfetti [43], low-quality lexical representations lead to comprehension difficulty because the lack of automatic and/or precise associations, either at the junction of orthography-phonology or phonology-semantics, which causes information necessary for integrating a word into its sentential context to be unavailable at the time when it is needed. Van Dyke et al. [40] reported that comprehension of subject and object relatives was much more related to verbal intelligence than to working memory [39]. The same may also be true for individuals with dyslexia, who are often reported to have lower verbal intelligence [25,41]. In summary, we expected individuals with dyslexia to show differences both in terms of comprehension and eye movements, and thus, the first goal of the study is to test whether these predictions hold for subject and object relatives. 

### 1.2. Psycholinguistic Theories–Relative Clauses

Several studies have established that sentences containing object relatives are more difficult to comprehend than sentences containing subject relatives [1,3,44]. The difficulty can be manipulated by several factors, such as animacy and semantic similarity of the noun phrases occurring in the sentence [1,8,45,46,47], as well as by the fact that object relatives are much less common than subject relatives [48]. According to Gibson’s [9] Syntactic Prediction Locality Theory (SPLT), which emphasises memory processes, it is predicted that while processing a sentence with a relative clause, more difficulty should arise at the relative clause verb (e.g., **passed** in a sentence like ‘The fisherman that the hiker passed carried the heavy gear’) [5,10]. On the other hand, a probabilistic expectation-based account (e.g., [4]), which focuses on experience- and frequency-based expectations, predict earlier difficulty at the relative clause noun (e.g., **hiker** in the previous example). These differential predictions are important for two reasons. The first is that the source of the processing difficulty is distinct. One class of theory assumes working memory demands are the key factor, while the other assumes that difficulty arises from a violation of predictive expectation. The second reason is that the theories make different predictions about where processing difficulty should be incurred. 

Eye movement studies on object and subject relatives have reported an increased number of regressions and longer reading times for object relatives compared to subject relatives [3,47,49]. Expanding on previous eye-tracking studies, Staub [44] reported, in a study that more closely resembled normal reading, that sentences with object relatives took longer to read than sentences with subject relatives. In particular, he showed elevated reading times at the relative verb and increased regressions from the relative noun. Based on this pattern, Staub concluded that both ‘classes’ of theories were partially correct (i.e., difficulty at the noun was in the form of increased regression, consistent with violation of expectation, and difficulty at the verb was in the form of elevated reading times, consistent with memory retrieval once the verb was encountered). 

To date, there has only been one study to examine the comprehension of subject and object relatives in dyslexia. Wiseheart, Altmann, Park and Lombardino [50] examined subject and object relatives in adults with and without dyslexia. Participants were shown a sentence and two images side-by-side on a computer screen, and they were asked to select the image that corresponded to the sentence. Wiseheart et al. [50] showed that dyslexic readers had poorer comprehension accuracy compared to the control group. Controls were 93% accurate on subject relatives and 97% on object relatives, while dyslexics were 84% accurate on subject relatives and 84% accurate on object relatives. Note that the pattern for the object relatives in controls was in the opposite direction of what is most commonly reported in the psycholinguistics literature. Wiseheart et al. [50] argued that dyslexics showed poorer comprehension accuracy compared to controls, as subject and object relatives place high demands on working memory and the individuals with dyslexia, in their sample, had lower working memory than did controls. This was further confirmed in an analysis in which working memory was covaried, as the effect of group was no longer significant. A key missing component in the Wiseheart et al. study was online-processing measures. Thus, we do not know whether/where dyslexic participants experienced online-processing difficulty, in addition to the offline comprehension impairments. 

### 1.3. Current Study

As mentioned in the opening paragraph, the main goals of the current study are (1) to investigate whether individuals with dyslexia have difficulty processing and comprehending subject and object relatives, and (2) to contribute to theoretical debates concerning both the source of processing difficulty associated with object relatives, and also, the location of processing difficulty. To investigate these goals, we monitored eye movements as participants read subject and object relatives, and we administered additional tasks to determine how individual differences in working memory [14] and verbal intelligence [41,51] were related to both online processing and offline comprehension.

Analyses focused on whether there were differences in the eye movement measures between participants with dyslexia and controls, and whether there were effects of verbal intelligence and working memory on comprehension and reading times. We expected participants with dyslexia to show poorer comprehension compared to controls, as well as to show differential eye movement patterns. More specifically, we expected to see longer reading times, more regressions and longer regression path durations in dyslexic participants in the key regions of the relative clause. Regarding the theoretical psycholinguistic debate, Gibson’s [9] SPLT predicts difficulty at the verb in an object relative, as there is a ‘storage cost’ that slows processing while the long-distance dependency is unresolved. In contrast, expectation-based theories (e.g., [4,8]) predict difficulty at the relative noun. Thus, we focused our eye movement analyses on the relative verb and relative noun in the relative clause [3]. If we find more processing difficulty at either the noun or the verb, then this would provide support for the theory that predicts difficulty at each location. Moreover, because we assessed individual differences in verbal intelligence and working memory, we were in a position to provide additional confirmatory evidence to support the underlying factors responsible for the processing difficulty associated with object relatives.

## 2. Materials and Methods

### 2.1. Participants

Fifty adults with dyslexia were recruited via advertisements and 50 undergraduate psychology students were tested as control participants (Information about statistical power is provided in the Supplementary Materials). Psychology students were recruited through the participant pool and received course credit. Dyslexic students were primarily recruited through disability liaison officers in different departments, as a function of being on the disability register at the university. Both groups were recruited from the campus of the University of East Anglia. All participants with dyslexia verified that they had a prior diagnostic assessment for dyslexia (by an educational psychologist or dyslexia specialist), prior to study enrolment. All were native speakers of British English with normal or corrected-to-normal vision. Dyslexics were reimbursed with £16 for their time. Demographic information about the two groups is provided in Table 2, as are the means for the individual differences variables. Table 3 shows the correlations between the demographic variables, the individual differences variables, and comprehension accuracy for subject and object relatives.

### 2.2. Standardised Measures

#### 2.2.1. Rapid Automatised Naming

All participants completed both a letter and a number RAN test [52] using the Comprehensive Test Of Phonological Processing (CTOPP 2). The RAN task requires participants to name a series of letters or numbers sequentially out loud as quickly and accurately as possible. The time taken to complete an array was recorded with a stopwatch. Participants completed one letter array for practice, and two served as the critical trials (i.e., one letter array and one number array). The score for each task was the total time that was needed to complete the task, with higher scores indicating worse performance. Each array consisted of four rows of nine items. Letters and numbers were presented in Arial font, and all items appeared on the same side of a white sheet of A4 paper. The standardised procedures of administration for this task were followed as described in the test manual. Independent samples *t*-tests revealed significantly longer naming times for the dyslexic group on both the letter and number array (see Table 2). The reliability of the CTOPP-2 subtests was demonstrated by average internal consistency that exceeds 0.80 [53].

#### 2.2.2. Working Memory 

A rotation span task was used as a measure of working memory, as it has been shown to assess both processing and storage functions [54,55]. Participants were required to look at a rotated letter and then verify whether the letter is facing in the correct direction or not (mirrored). After each letter, participants were presented with an isolated arrow which was either long or short and could be facing eight different directions (0°–360°). The position and length of the arrows presented needed to be recalled at the end of the set. The task consisted of 15 trials (six each of list length 2 and three each of list lengths 3–5) and in total 48 arrow-storage pairs [55]. The rotation span task was developed by Engle’s Working Memory Laboratory, and reported reliability ranging between 0.67 and 0.77 for the rotation span [56].

#### 2.2.3. Verbal Intelligence 

Verbal intelligence was measured by the following subtests of the fourth edition of the Wechsler Adult Intelligence Scale (WAIS-IV) [57]: vocabulary, comprehension and similarities. In the comprehension task, participants were required to respond to questions about general concepts (e.g., reasons to protect endangered species). Vocabulary requires participants to provide the definitions of words and measures the degree to which one has learned and is able to express meanings verbally. Similarities require participants to describe how two words are similar, with the more difficult items typically describing the opposite ends of a ‘unifying continuum’. The similarities subtest measures abstract verbal reasoning [39]. For all subtests, higher values correspond to higher verbal intelligence and the score for each of these tasks was the total number of items that the participants could identify accurately. The standardised procedures of administration for these subtests were followed as described in the test manual. With respect to the reliability of the WAIS-IV, the manual reports average internal reliability coefficients for subtests that range from 0.78 to 0.94 [58].

### 2.3. Sentence Processing

To investigate subject and object relatives, we used 20 sentences based on the items in Traxler et al. [3]. Each participant read ten sentences containing object-relative clauses and ten containing subject-relative clauses. Items were rotated in a Latin Square Design. All 20 critical items were rotated across two counterbalance lists, with object relatives changing to subject relatives and vice versa (see Table 1). Ten sentences with relative clauses required a ‘yes’ response and ten required a ‘no’ response. All questions for sentences with relative clauses rotated across four counterbalance lists, with changing accordingly to require a ‘yes’ or ‘no’ response and vice versa for each version of every item.

Participants also read 120 filler sentences. All filler sentences were grammatically correct. They consisted of five sets of 16 sentences. The first set was subordinate-main structures in which the subordinate clause was transitive. The second set was the main-subordinate sentences. The third set was transitive sentences containing a relative clause at the end of the sentence. The fourth set was the transitive sentences that contained an embedded relative clause that modified the subject noun phrase. The fifth set was the coordination structures, in which two transitive sentences were conjoined with ‘and’. Half of these had a comma between ‘and’ and the preceding word and half did not. In addition, there were also 20 active and passive sentences. Half of these were implausible, and half were plausible. There were also 20 sentences containing a subject or object relative clause following the main clause. Therefore, each participant read 140 sentences in total. Fifty-eight filler questions required a ‘yes’ response and 62 required a ‘no’ response.

### 2.4. Apparatus

Eye movements were recorded with an SR Research Ltd. EyeLink 1000 eye-tracker which records the position of the reader’s eye every millisecond. Head movements were minimised with a chin rest. Viewing distance was 70 cm from eyes to a 45-cm computer monitor, and at this distance, 1.0° of visual angle subtended 1.22 cm. This apparatus allows recording of eye movements through a camera with an infrared tracking system. Eye movements were recorded from the right eye. The sentences were presented in 12 pt. Arial black font on a white background.

### 2.5. Design and Procedure

For the sentence processing task, the design was a 2 × 2 (Type × Group) mixed design, in which ‘type’ was within subjects, and ‘group’ was between subjects. Participants completed three practice trials, 20 experimental trials and 120 fillers. Trials were presented in a random order for each participant.

Participants were provided with a set of instructions that detailed the experimental procedure. They were then seated at the eye tracker and asked to respond to on-screen instructions using the keyboard. At the beginning of each trial, a message appeared asking the participant to press a button when they were ready to continue. After the participant pressed the button, they were required to fixate a drift-correction dot. The experimenter then initiated the trial. The sentence appeared after 500 ms, and the initial letter of each sentence was in the same position, in terms of x and y coordinates, as the drift correction dot (i.e., on the left edge of the monitor and centred vertically).

The entire sentence was presented on a single line on the screen. The participant read the sentence silently and then pressed the spacebar on the keyboard. Following a delay of 500 ms, an arithmetic problem (either addition or subtraction) appeared on the screen (e.g., 45 + 67 = 112). The problem was presented for 3000 ms and was followed by a screen prompting the participant to press the green button on the keyboard if the solution was correct, or the red button if it was incorrect. After participants read the sentence, they were asked a comprehension question, such as ‘Did the hiker pass the fisherman?’. For the reliability of the sentence processing task, we computed split-half reliabilities. Because there were ten items in each of the within-subject conditions, we used Spearman–Brown prophecy formula-corrected coefficients [59,60]. The mean reliability was *α* = 0.34.

The purpose of the additional arithmetic problem was to assess the representation that comprehenders generated of the sentences, without allowing them to have direct access to the sentence. We expected that the presence of the mathematical problem would clear the immediate contents of working memory, therefore resulting in the participants responding to the comprehension questions on the basis of a more long-term representation/trace of the sentence.

The testing session for each participant lasted approximately 2 h, with several breaks included between tasks to avoid fatigue. The tests were delivered in the following order for each participant: vocabulary, rotation span, comprehension, sentence processing, RAN digits, RAN letters and similarities.

### 2.6. Data Screening and Analysis

In order to keep the analyses as straightforward as possible we submitted the verbal intelligence subtests to a factor analysis (principal components extraction) in which we saved the retained factor(s) as variable. The results of the factor analysis showed only one factor (eigenvalue = 1.81, accounting for ~60% of the total variance). The factor loadings were all significant and relatively uniform (vocabulary = 0.84, comprehension = 0.76 and similarities = 0.72). We used this composite (or latent) variable in our analyses examining ‘individual differences in verbal intelligence’. 

We analysed the comprehension and eye movement data using standard ANOVAs with subjects (*F1*) and items (*F2*) as random effects (Companion analyses using Linear Mixed Effects models are presented in the Supplementary Materials for specialist readers). First pass reading time is the sum of all fixations on a word from when a reader first enters a region to when they leave that region either forward or backward. Total reading time is the sum of all fixations on a word. Regressions out of an interest area are the sum of all right-to-left eye movements to previously read word. Regression path duration is the sum of all fixations from the time the eyes first enter a region until they move beyond that region in a forward direction. We analysed data from two main regions of interest, which included the relative clause verb and the relative noun (see Table 1, for examples). We first report the comprehension results, and second, the eye movements. To assess verbal intelligence and working memory, we conducted two additional ANCOVAs in which each variable was co-varied separately.

## 3. Results

### 3.1. Comprehension Accuracy

**Figure 1:**
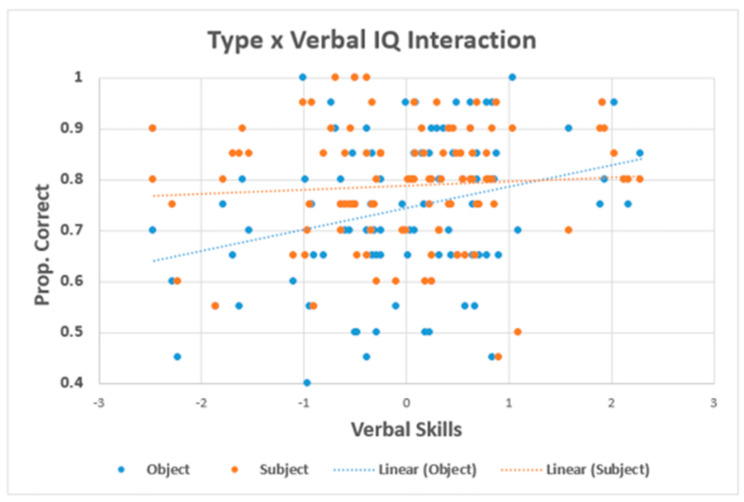
Mean comprehension accuracy. Error bars show the standard error of the mean.

**Figure 2:**
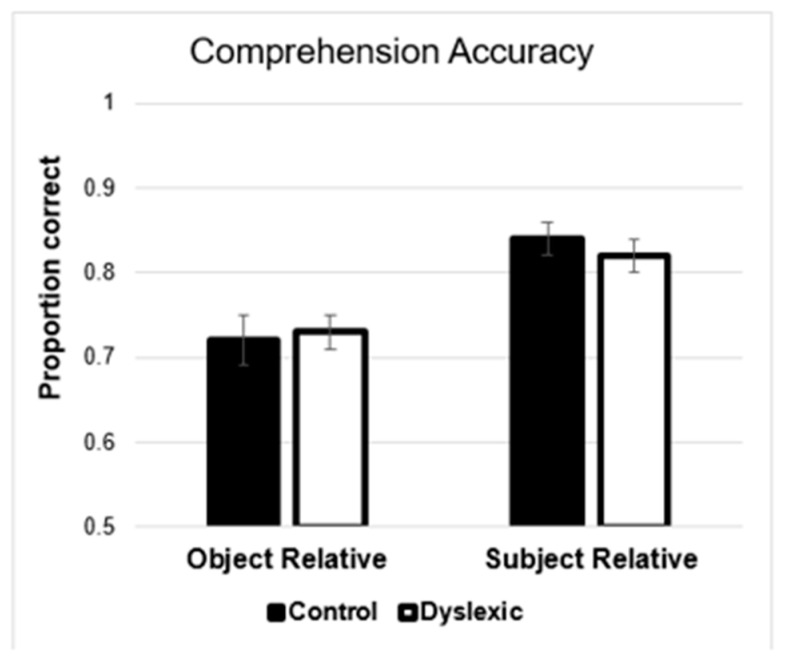
Sentence ‘Type’ by verbal intelligence interaction.

The mean comprehension accuracies are presented in Figure 1, and the results of the inferential analyses are presented in Table 4. Results showed a main effect of type, in which the subject relatives had higher comprehension than did object relatives. When verbal intelligence was included in the model, it produced a main effect and interacted with type. The form of the interaction is shown in Figure 2. As can be seen, verbal intelligence was positively related to comprehension of object relatives, such that, individuals with lower verbal intelligence showed many more incorrect responses for object relatives. In contrast, with subject relatives, there was not much of an effect of verbal intelligence. When working memory was included in the model, it produced a significant main effect and the main effect of type remained significant (although the effect size was approximately four times smaller). This pattern of results suggests overlapping variance between individual differences in working memory and comprehension. That is, when variance in working memory was removed, then the difference in comprehension between subject and object relatives was substantially reduced. To ensure the direction and the strength of the relationship between working memory and comprehension, we ran the correlations between working memory and subject relatives, and between working memory and object relatives. In both cases, the relationship was positive, and for the subject relatives, the correlation was significant (r = 0.20, *p* < 0.05). For object relatives, the correlation was similar (r = 0.17, *p* = 0.098) but not significant. In the comprehension, there was no effect of group (i.e., control vs. dyslexia), which suggests that the individuals with dyslexia are not worse at comprehending these particular types of sentences (cf. [50]). 

### 3.2. Eye Movements–Relative Verb

#### 3.2.1. Reading Times

The means for the eye movement measures are presented in Table 5, and the results of the inferential analyses are presented in Table 6. Results showed a largely consistent pattern for both first pass reading times and total reading times. There were main effects of type and group, in which object relatives had higher reading times than did subject relatives, and likewise, individuals with dyslexia had higher reading times than did controls. The mean difference between subject and object relatives was 38 msec on first pass and 141 msec on total reading time. For group, the mean difference between controls and dyslexics was 44 msec on first pass reading times and 291 msec on total reading times. When verbal intelligence was included, the same pattern of results emerged, and verbal intelligence was not significant and did not interact with sentence type. When working memory was included in the model, the main effect of type remained significant only for the total reading times and the main effect of group remained unchanged in both measures. What this pattern tells us, similar to comprehension accuracy, is that when variance in working memory is removed, the processing difficulty between subject and object relatives disappeared for first pass reading times (i.e., there is overlapping variance between reading times and individual differences in working memory).

#### 3.2.2. Regressions

For regressions out of the relative verb, there were no significant effects. Across all trials, we observed that there were approximately one-in-four to one-in-three trials with a regression. For regression path durations, results showed that both the main effect of type and group were significant and remained significant with the inclusion of both covariates. Object relatives had approximately 79 msec longer regression paths than did subject relatives, and dyslexics had approximately 179 msec longer regression paths than did controls. 

We also observed a main effect of verbal intelligence, and the pattern was such that individuals with higher verbal intelligence had shorter regression path durations. The correlation between object relatives and verbal intelligence was marginally significant (r = 0.19, *p* = 0.06) and for subject relatives it was not significant (r = 0.11, *p* = 0.26).

### 3.3. Eye Movements–Relative Noun

#### 3.3.1. Reading Times

The means for the eye movement measures are presented in Table 5 and the results of the inferential analyses are presented in Table 7. Results showed some similarities to the patterns that were observed at the relative verb, this is especially true of the total reading times, which were identical. In contrast, in first pass reading time, there was no significant effect of group, but there was a consistent group effect on total reading times. Participants with dyslexia had approximately 200 msec longer total reading times than did controls, and this effect remained significant with the inclusion of both verbal intelligence and working memory. Similar to the results at the relative verb, the main effect of type was not significant when working memory was included in the model, again suggesting some overlapping variance between individual differences in working memory and the difficulty incurred in processing object relatives compared to subject relatives.

#### 3.3.2. Regressions

For regressions out of the relative noun, there was only a significant effect of type, regressions were more frequent from object relatives compared to subject relatives. This effect held when verbal intelligence was included in the model but not working memory. Across all trials, we observed slightly fewer regressions from the relative noun. In this case, there were approximately one-in-five to one-in-four trials with a regression. The pattern of results in regression path durations was similar to total reading times at the relative noun and first pass and total reading times at the relative verb. There were significant main effects of type and group. Group was robust to the inclusion of both covariates and the same was the case for the main effect of type.

#### 3.3.3. Relationship between Online and Offline Measures

The correlations between eye movements, individual difference measures and comprehension are presented in Table 8. They revealed only one significant correlation between eye movements and comprehension. The total reading time on the relative verb (in subject relative sentences) correlated with comprehension accuracy. For object relatives there were no significant correlations, and in fact, there were two that were in the opposite direction of what would be expected by more processing effect resulting in better comprehension. Those two negative correlations occurred at the relative noun for regressions out (0.13) and regression path duration (0.16). We think these two results partially support the speculations made by Staub about regressions being linked with parsing integration failures, and recall that Staub did find increased regressions from the relative noun. Therefore, there are trends in our data that partially support speculations about regressions and parsing failures. The other important points from our correlational data are (1) that dyslexia is strongly related to eye movement behaviour and the direction of that relationship is for individuals with dyslexia to show elevated reading times, and (2) individual differences in verbal intelligence and working memory are most strongly related to regression path durations in object relatives.

## 4. Discussion

In this study, we examined how dyslexic and non-dyslexic adults comprehend and process sentences with complex syntax, and specifically, sentences that contain subject- and object-relative clauses. We were interested in whether individuals with dyslexia show deficits in comprehension and how their eye movement behaviour differed from control participants. We also explored the impact of two individual differences variables (i.e., working memory and verbal intelligence) as potential key individual difference variables in the processing of subject- and object-relative clauses. A second goal of the study was to contribute to theoretical debates on both the location and cause of processing difficulty associated with object relatives. Here the choice of dyslexia was key, as individuals with dyslexia often have lower working memory, and in one recent study, were reported to have deficits in linguistic prediction [16]. Thus, individuals with dyslexia are assumed to have deficits in the two ‘sources’ of processing difficulty proposed by the competing psycholinguistic theories (e.g., [4 vs. 9]). In this case, the goal was to use a clinical population to inform theoretical debate. 

### 4.1. Processing Relative Clauses in Dyslexia 

To summarise our main findings with respect to dyslexia, we found that individuals with dyslexia had similar comprehension accuracy compared to controls. Despite the fact that dyslexics showed similar comprehension to controls, they spent significantly longer reading the sentences. More specifically, our results with respect to eye movements showed that the dyslexics showed longer first pass reading times, longer total reading times and longer regression path durations. These findings occurred for both regions of interest, except that the group difference in first pass reading times was not significant at the relative noun. In addition, there were no significant group effects in terms of regressions out of the regions of interest, and group did not interact with any of the other variables (i.e., type, verbal intelligence, or working memory). The lack of interactions is consistent with most of the other studies from our lab. In short, we tend to observe a robust main effect of group and no interaction(s). This suggests that dyslexia does not result in over- or under-additive effects on various psycholinguistic manipulations. In the current study, individuals with dyslexia spent longer in reading than did controls, and ultimately, achieved very similar performance in terms of comprehension accuracy. Finally, in this study, neither of the individual difference variables were related to the group effect (i.e., dyslexia appeared to have an effect on the time spent in reading, independent of the individual differences in verbal intelligence and working memory).

In the field of psycholinguistics, the vast majority of research on the processing of subject- and object-relative clauses has been conducted on typically developed samples (e.g., [9,45,61]). In the Introduction, we reviewed the results from a similar paper that examined the comprehension of subject and object relatives in dyslexia (i.e., [50]). Our results were inconsistent with that study in two main ways. The first is that we did not find differences in terms of comprehension, and the second is that in Wiseheart et al. dyslexia status and working memory shared more variance (i.e., covarying working memory eliminated the group effect on comprehension). There are several differences between the two studies that may account for the discrepancies. The most important difference is the experimental paradigm. Wiseheart et al. used a picture-sentence verification task in which two pictures were on the screen with the sentence. In short, in Wiseheart et al. [50], the comprehension decision was made when the sentence was still visible. In contrast, in our paradigm there was an intervening maths problem and participants were answering very specific comprehension questions regarding thematic roles and the association of specific nouns with specific verbs. As a result of the increased difficulty of our task, overall comprehension accuracy was approximately 15% lower in the current study.

Another difference concerns the sample. In our study participants were all university students, and in Wiseheart et al.’s participants were younger and had significantly lower working memory scores. The age discrepancy is important because our participants may have more exposure to complex syntax given their enrolment in higher education. Due to the multiple differences, it is very difficult to make concrete conclusions about comprehension deficits in subject and object relatives. What our results do clearly show is significant differences in online processing (i.e., dyslexics required more processing time to achieve a similar comprehension level). Careful consideration of the participant sample and the experimental paradigm will be important in future studies.

### 4.2. Eye Movements in Relative Clause Region

Recall that Staub [44] reported a dissociation in the eye movements occurring in the relative noun and relative verb. More specifically, he found an increase in the number of regressive eye movements but no increase in first pass reading times at the noun, and elevated first pass reading times but not an increase in the number of regressive eye movements at the verb [12,44]. On the basis of this dissociation, Staub concluded that both theoretical accounts (i.e., memory-based vs. expectation-based) were partially correct and both contribute to the processing of relative clauses (e.g., [1,8,11,13,62,63]). Moreover, Staub speculated that the dissociation in eye movement patterns may reflect different underlying processing effects. An increase in fixation durations reflects processing difficulty that eventually succeeds, and an increase in regressions reflects processing difficulty that has failed [3,46,47].

Comparing Staub’s findings to ours, reveals some striking similarities, but also some differences. We begin with the similarities. At the relative verb, we found effects of type on all three fixation ‘duration’ measures (i.e., first pass reading time, total reading time, and regression path duration), and there were no significant effects on regressions out of the relative verb. Related to fixation durations, all of the measures showed a clear pattern suggesting that processing difficulty was more affected by individual differences in working memory. When working memory was included in the model, the effect sizes of type were substantially reduced, especially for first pass and total reading times. In contrast, when verbal intelligence was included the effect sizes remained unchanged. The differences between our study and Staub primarily occurred on the relative noun region. However, the results at the relative noun did show some similarities to Staub. Recall that processing difficulty was predicted at the relative noun to be due to experience-based factors and surprisal (i.e., that object relatives are more infrequent than subject relatives and thus, less expected in terms of parsing expectations/predictions) [44]. We found an increased number of regressions from the relative noun (consistent with Staub), but also elevated fixation durations for all three duration-based measures (inconsistent with Staub).

The key finding of our study concerning processing difficulty at the relative noun, which is particularly difficult to reconcile with Staub’s study is that for first pass reading times and total reading times the effect size of type was, again, substantially reduced with the inclusion of working memory in the model. For these two dependent measures, the effect size was reduced by two-thirds once individual differences in working memory were controlled. Thus, in our data, processing difficulty at the noun also seemed to be linked to individual differences in working memory. Several other points are worth mentioning. The first is that like Staub, we observed increased first pass reading time on the relative noun in subject relatives, which was in the opposite directions to all other findings with respect to eye movement measures (i.e., the subject relatives had higher reading times than did object relatives on the relative noun). The second is that the proportion of trials with a regression at the relative noun in the current study was much lower. Staub [44] reported 0.40 in object relatives and 0.16 in subject relatives. In contrast, we found 0.23 in object relatives and 0.17 in subject relatives (for our controls). Individuals with dyslexia, in the current study, were slightly higher for both. Therefore, we did not observe nearly as high a rate of regressions from the relative noun, despite the difference being statistically significant. The third point is that we observed much longer regression path durations, for controls and especially for individuals with dyslexia.

In general, we feel that the most important take home message from the current study, with respect to eye movements and the comparisons to Staub [44], is that processing difficulty was more related to individual differences in working memory. Moreover, individuals with dyslexia showed even longer reading times compared to controls, and those differences were not accounted for by individual differences in working memory or verbal intelligence. Thus, on the basis of our findings, we believe that much more of the processing difficulty incurred with object relatives is due to memory-based processes, and in particular holding the extracted constituent in memory rather than retrieving the constituent at the moment the relative verb in encountered.

### 4.3. Limitations and Future Directions

One of the main strengths of this study is the fact that we assessed the performance of a large number of participants on a variety of different tasks. However, because our sample of dyslexics was recruited through a university, they were quite high functioning. This is potentially problematic because often individuals with dyslexia do not go on to higher education. It remains to future work to determine if a sample of community-recruited dyslexics achieves similar performance in terms of comprehension accuracy and individual differences. Furthermore, our sample of dyslexics was potentially atypical, so far as they had similar working memory and verbal intelligence as the controls. To assess working memory, we used a rotation span task, which did not include any literacy or reading components in order to avoid any additional difficulties for participants with dyslexia. However, we only had a single measure. In future, we would recommend using multiple measures of working memory, and also, including some that have linguistic component (e.g., reading span). Future work should also investigate the processing of subject and object relatives using some of the manipulations that have been investigated in the psycholinguistic literature (e.g., animate and inanimate nouns), which would allow future studies to examine how semantic issues affect dyslexic readers’ comprehension of relative clause sentences [45]. We would also recommend for future research to include standardised reading, spelling or phonological awareness assessments as additional measures of participants’ dyslexia diagnosis. Moreover, we suggest that dyslexia should be examined across the lifespan, which calls for further research on children and adolescents in order to investigate the processing of sentences prior to adulthood, as well as during the critical period of reading acquisition.

## 5. Conclusions

This study aimed first to investigate processing and comprehension of sentences that contain relative clauses in individuals with dyslexia. We found three main findings with respect to this aim: individuals with dyslexia (1) achieved similar performance in terms of comprehension accuracy, (2) showed significantly longer reading times and (3) the effect of dyslexia was robust even when individual differences in verbal intelligence and working memory were controlled. The second main aim of the study was to contribute to the psycholinguistic debate concerning where and why processing difficulty occurs in object relatives as compared to subject relatives, and this aim focused exclusively on the eye movement results. Here our data were very clearly linked to individual differences in working memory, such that when variance in working memory was removed the differences between subject and object relatives was no longer significant. Moreover, working memory also accounted for the subject–object difference even at the relative noun, which refutes prior claims about processing difficulty at this word being linked to violations of expectations. Thus, overall, our eye movement and individual differences analysis supports theories of processing difficulty that assume difficulty is linked with memory-based processing (e.g., [9]) rather than surprisal [4,5]. 

## Figures and Tables

**Table 1:** Example stimuli showing object- and subject-relative clauses, and comprehension questions.

**Object Relative**
The fisherman that the | **hiker** | **passed** | carried heavy gear.
Comprehension Questions
Did the hiker pass the fisherman? (correct answer = Yes)
Did the fisherman pass the hiker? (correct answer = No)
**Subject Relative**
The fisherman that | **passed** | the | **hiker** | carried heavy gear.
Comprehension Questions
Did the fisherman pass the hiker? (correct answer = Yes)
Did the hiker pass the fisherman? (correct answer = No)

Note: Bolded words show key regions of interest (hiker = relative noun, passed = relative verb). Words were not bolded in the experiment.

**Table 2:** Means and standard deviations for demographic variables, the Rapid Automatised Naming task and the individual differences variables.

	Controls (*n* = 50)	Dyslexia (*n* = 50)	*t*-Value
Variable	Mean (SD)	Mean (SD)	
Age (years)	20.31 (1.22)	21.7 (2.67)	*t*(98) = 3.34 ***
Gender (% male)	8	34	*t*(98) = 3.33 ***
Handedness (% left)	12	10	*t*(98) = −0.317
RAN Letters (seconds)	12.46 (2.59)	16.50 (6.20)	*t*(98) = 4.25 ***
RAN Numbers (seconds)	11.44 (2.43)	15.26 (5.29)	*t*(98) = 4.64 ***
Similarities	93.5 (8.65)	98.8 (11.76)	*t*(98) =−2.57 *
Vocabulary	99.9 (9.18)	101.3 (9.02)	*t*(98) = −0.77
Comprehension	93.5 (10.70)	94.3 (9.31)	*t*(98) =0.40
Verbal Skills (latent)	0.152 (0.98)	0.152 (1.00)	*t*(98) = −1.53
Rotation Span	17.7 (7.23)	16.9 (8.04)	*t*(98) = 0.51

Note: * *p* < 0.05, *** *p* < 0.001.

**Table 3:** Correlations between demographics, individual difference variables and comprehension.

	1	2	3	4	5	6	7	8	9
1. Age	-	0.35 **	0.32 **	−0.18	−0.17	0.16	0.04	0.1	0.13
2. Gender		-	0.32 **	−0.24 *	−0.19	0.13	0.30 **	0.11	0.1
3. Dyslexia Status			-	0.42 **	0.40 **	−0.05	0.15	0.05	−0.07
4. RAN Numbers				-	0.92 **	0.40 **	−0.05	−0.18	−0.11
5. RAN Letters					-	0.31 **	−0.07	−0.16	−0.05
6. Rotation Span						-	−0.04	0.17	0.18
7. Verbal Intelligence							-	0.30 **	0.04
8. Object Relative								-	0.20 *
9. Subject Relative									-

Note: * *p* < 0.05, ** *p* < 0.01. Gender coded 0 = female and 1 = male. Dyslexia coded 1 = dyslexic and 0 = control.

**Table 4:** Inferential results for comprehension accuracy.

**2 × 2 (Type × Group)**	
Type	*F*(1,98) = 29.69, *p* < 0.001, η_p_^2^ = 0.23
Group	*F*(1,98) = 0.002, *p* = 0.97
Type × Group	*F*(1,98) = 0.78, *p* = 0.38
**ANCOVA—with Verbal IQ**	
Type	*F*(1,97) = 31.16, *p* < 0.001, η_p_^2^ = 0.24
Group	*F*(1,97) = 0.18, *p* = 0.67
Verbal IQ	*F*(1,97) = 6.23, *p* < 0.05, η_p_^2^ = 0.06
Type × Group	*F*(1,97) = 0.28, *p* = 0.60
Type × Verbal IQ	*F*(1,97) = 5.84, *p* < 0.05, η_p_^2^ = 0.06
**ANCOVA—with WM**	
Type	*F*(1,97) = 6.18, *p* < 0.05, η_p_^2^ = 0.06
Group	*F*(1,97) = 0.01, *p* = 0.94
Working Memory	*F*(1,97) = 4.98, *p* < 0.05, η_p_^2^ = 0.05
Type × Group	*F*(1,97) = 0.80, *p* = 0.37
Type × Working Memory	*F*(1,97) = 0.12, *p* = 0.73

**Table 5:** Mean reading times clause by group and experimental condition-relative verb.

First Pass RT		Total RT		Reg. Out		Reg. Path		
	*M*	*SD*	*M*	*SD*	*SD*	*SD*	*M*	*SD*
**Relative Verb**								
Controls								
OR centre	320.5	73.5	867.2	299.1	0.24	0.18	597.9	310.3
SR centre	291.9	61.1	703.8	262.9	0.26	0.16	504.4	195.5
Dyslexics								
OR centre	374.6	110.1	1134.9	492.5	0.28	0.14	762.5	340.2
SR centre	326.5	95.3	1015.6	465.9	0.32	0.16	696.8	311
**Relative Noun**								
Controls								
OR centre	257.2	49.4	655.5	281.6	0.23	0.16	474.1	182.6
SR centre	280.9	75.5	524.2	165.1	0.17	0.17	445.8	221.7
Dyslexics								
OR centre	255	67.3	820.6	460.6	0.28	0.17	668.2	361.7
SR centre	300.1	82.3	760.9	341.6	0.21	0.13	593.6	307.7

**Table 6:** Mixed ANCOVA analysis for eye movement measures for the relative verb.

	First Pass RT	Total RT	Reg. Out	Reg. Path
**2 × 2 (Type × Group)**				
Type	*F*(1,98) = 15.10, *p* < 0.001, (0.13) ^a^	*F*(1,98) = 19.18, *p* < 0.001, (0.16) ^a^	*F*(1,98) = 2.16, *p* = 0.15	*F*(1,98) = 7.45, *p* < 0.01, (0.07) ^a^
Group	*F*(1,98) = 9.56, *p* < 0.01, (0.09) ^a^	*F*(1,98) = 16.33, *p* < 0.001, (0.14) ^a^	*F*(1,98) = 3.26, *p* = 0.07	*F*(1,98) = 12.16, *p* < 0.01, (0.11) ^a^
Type × Group	*F*(1,98) = 0.97, *p* = 0.33	*F*(1,98) = 0.47, *p* = 0.50	*F*(1,98) = 0.61, *p* = 0.44	*F*(1,98) = 0.23, *p* = 0.64
**ANCOVA—with Verbal IQ**				
Type	*F*(1,97) = 15.08, *p* < 0.001, (0.14) ^a^	*F*(1,97) = 18.98, *p* < 0.001, (0.16) ^a^	*F*(1,97) = 2.15, *p* = 0.15	*F*(1,97) = 7.45, *p* < 0.01, (0.07) ^a^
Group	*F*(1,97) = 9.98, *p* < 0.01, (0.09) ^a^	*F*(1,97) = 16.03, *p* < 0.001, (0.14) ^a^	*F*(1,97) = 3.49, *p* = 0.07	*F*(1,97) = 15.28, *p* < 0.001, (0.14) ^a^
Verbal IQ	*F*(1,97) = 0.53, *p* = 0.47	*F*(1,97) = 0.04, *p* = 0.85	*F*(1,97) = 0.34, *p* = 0.56	*F*(1,97) = 6.04, *p* < 0.05, (0.06)
Type × Group	*F*(1,97) = 0.69, *p* = 0.41	*F*(1,97) = 0.42, *p* = 0.52	*F*(1,97) = 0.39, *p* = 0.54	*F*(1,97) = 1.00, *p* = 0.76
Type × Verbal IQ	*F*(1,97) = 0.89, *p* = 0.35	*F*(1,97) = 0.02, *p* = 0.88	*F*(1,97) = 0.92, *p* = 0.34	*F*(1,97) = 1.07, *p* = 0.31
**ANCOVA—with WM**				
Type	*F*(1,97) = 3.08, *p* = 0.08	*F*(1,97) = 6.01, *p* < 0.05, (0.06) ^a^	*F*(1,97) = 0.07, *p* = 0.79	*F*(1,97) = 8.07, *p* < 0.01, (0.08)
Group	*F*(1,97) = 9.20, *p* < 0.01, (0.09)	*F*(1,97) = 16.04, *p* < 0.001, (0.14) ^a^	*F*(1,97) = 3.23, *p* = 0.07	*F*(1,97) = 11.76, *p* < 0.01, (0.11)
Working Memory	*F*(1,97) = 1.42, *p* = 0.24	*F*(1,97) = 0.06, *p* = 0.81	*F*(1,97) = 0.00, *p* = 0.97	*F*(1,97) = 1.28, *p* = 0.26
Type × Group	*F*(1,97) = 0.94, *p* = 0.34	*F*(1,97) = 0.52, *p* = 0.47	*F*(1,97) = 0.68, *p* = 0.41	*F*(1,97) = 0.34, *p* = 0.56
Type × Working Memory	*F*(1,97) = 0.05, *p* = 0.82	*F*(1,97) = 0.59, *p* = 0.45	*F*(1,97) = 0.88, *p* = 0.35	*F*(1,97) = 3.58, *p* = 0.06

Note: Effect sizes η_p_^2^ are reported in parentheses. ^a^ indicates significant in F2 item analysis (see Supplementary Materials, Table S1).

**Table 7:** Mixed ANCOVA analysis for eye movement measures for the relative noun.

	First Pass RT	Total RT	Reg. Out	Reg. Path
**2 × 2 (Type × Group)**				
Type	*F*(1,98) = 24.57, *p* < 0.001, (0.20) ^a^	*F*(1,98) = 13.30, *p* < 0.001, (0.12) ^a^	*F*(1,98) = 9.81, *p* < 0.01, (0.09) ^a^	*F*(1,98) = 4.08, *p* < 0.05, (0.04)
Group	*F*(1,98) = 0.50, *p* = 0.48	*F*(1,98) = 10.70, *p* < 0.01, (0.10) ^a^	*F*(1,98) = 2.59, *p* = 0.11	*F*(1,98) = 12.03, *p* < 0.01, (0.11) ^a^
Type × Group	*F*(1,98) = 2.38, *p* = 0.13	*F*(1,98) = 1.87, *p* = 0.18	*F*(1,98) = 0.02, *p* = 0.90	*F*(1,98) = 0.83, *p* = 0.37
**ANCOVA—with Verbal IQ**				
Type	*F*(1,97) = 24.53, *p* < 0.001, (0.20) ^a^	*F*(1,97) = 13.24, *p* < 0.001, (0.12) ^a^	*F*(1,98) = 9.81, *p* < 0.01, (0.09) ^a^	*F*(1,97) = 4.05, *p* < 0.05, (0.04)
Group	*F*(1,97) = 0.55, *p* = 0.46	*F*(1,97) = 10.45, *p* < 0.01, (0.10)	*F*(1,97) = 2.67, *p* = 0.11	*F*(1,97) = 13.74, *p* < 0.001, (0.12) ^a^
Verbal IQ	*F*(1,97) = 0.09, *p* = 0.77	*F*(1,97) = 0.01, *p* = 0.91	*F*(1,97) = 0.12, *p* = 0.74	*F*(1,97) = 2.67, *p* = 0.11
Type × Group	*F*(1,97) = 1.91, *p* = 0.17	*F*(1,97) = 1.52, *p* = 0.22	*F*(1,97) = 0.00, *p* = 0.99	*F*(1,97) = 0.65, *p* = 0.42
Type × Verbal IQ	*F(1,97) = 0.87, *p* = 0.35*	*F*(1,97) = 0.58, *p* = 0.46	*F*(1,97) = 0.95, *p* = 0.33	
**ANCOVA—with WM**				
Type	*F*(1,97) = 7.18, *p* < 0.01, (0.07) ^a^	*F*(1,97) = 4.37, *p* < 0.05, (0.04) ^a^	*F*(1,97) = 6.41, *p* < 0.05, (0.06)	*F*(1,97) = 4.04, *p* < 0.05, (0.04)
Group	*F*(1,97) = 0.42, *p* = 0.52	*F*(1,97) = 10.42, *p* < 0.01, (0.10) ^a^	*F*(1,97) = 2.43, *p* = 0.12	*F*(1,97) = 11.64, *p* < 0.01, (0.11)
Working Memory	*F*(1,97) = 1.14, *p* = 0.29	*F*(1,97) = 0.25, *p* = 0.62	*F*(1,97) = 0.87, *p* = 0.35	*F*(1,97) = 3.42, *p* = 0.07
Type × Group	*F*(1,97) = 2.25, *p* = 0.14	*F*(1,97) = 1.95, *p* = 0.17	*F*(1,97) = 0.00, *p* = 0.95	*F*(1,97) = 0.71, *p* = 0.40
Type × Working Memory	*F*(1,97) = 0.58, *p* = 0.45	*F*(1,97) = 0.48, *p* = 0.49	*F*(1,97) = 1.93, *p* = 0.17	*F*(1,97) = 1.71, *p* = 0.19

Note: Effect sizes η_p_^2^ are reported in parentheses. ^a^ indicates significant in F2 item analysis (see Supplementary Materials, Table S2).

**Table 8:** Bivariate correlations between individual differences variables, comprehension and eye movement measures.

		Object Relative				Subject Relative		
	First Pass	Total RT	Reg. Out	Reg. Path	First Pass	Total RT	Reg. Out	Reg. Path
**Relative Verb**								
Dysle×ia Status	0.28 **	0.32 **	0.11	0.25 *	0.21 *	0.38 **	0.19	0.35 **
Verbal Intelligence	0.04	0.03	−0.09	−0.19	−0.09	0.05	0.04	−0.11
Working Memory	−0.11	−0.07	−0.06	−0.18	−0.1	−0.01	0.05	−0.02
Comp. Object	0.14	0.09	0.02	0.01				
Comp. Subject					−0.07	0.23 *	0.11	0.01
**Relative Noun**								
Dysle×ia Status	−0.02	0.21 *	0.13	0.32 **	0.120	0.40 **	0.13	0.027 **
Verbal Intelligence	−0.09	0	0.05	−0.06	0.04	0.08	−0.07	−0.13
Working Memory	−0.06	−0.07	−0.16	0.22 *	−0.12	−0.04	0.01	−0.12
Comp. Object	−0.05	0.06	−0.13	−0.16				
Comp. Subject					0.07	0.16	0.14	0.11

Note: * *p* < 0.05, ** *p* < 0.01.

## Data Availability

The data presented in this study are available on request from the corresponding author.

## Supplementary Materials

The following are available online at https://www.mdpi.com/article/10.3390/brainsci11070915/s1, Table S1: Mixed ANCOVA item analysis for eye movement measures for the relative verb, Table S2: Mixed ANCOVA item analysis for eye movement measures for the relative noun.
