# Supplementary material for: Comprehension and Eye Movements in the Processing of Subject- and Object-Relative Clauses: Evidence from Dyslexia and Individual Differences †
Source: Brain Sci. 2021 Jul 10;11(7):915. doi: 10.3390/brainsci11070915 (PMC8307189; doi:10.3390/brainsci11070915)
Supplement: Supplementary file 1 [file brainsci-11-00915-s001.zip › brainsci-1269627-supplementary.pdf]

Supplementary Materials

Section A: Power Analysis

With respect to statistical power, using from comprehension results based on Wiseheart et al. (i.e. control = .95 and dyslexia = .84, SD= .14), and considering an alpha of .05 and power of .80, suggests a sample size of only 26. Conversely, if we calculate estimated power based on sample sizes of 50, we get estimated power of .98.

Section B: R Analysis

Internal Replication: The code and data (Rmd file and two csv files) for these analyses are available upon request. All of the following converged without a singularity warning. If singularity warnings did occur, we always removed "group" from items first, and then if that still gave a singularity error we removed "type" from subject. Analyses contained below begin with the relative noun region of interest (total reading time, first pass reading time, regressions, and regression path duration). The second set of analysis are the relative verb (total reading time, first pass reading time, regressions, and regression path duration).

June 28, 2021

```
knitr::opts_chunk$set(echo = TRUE)


suppressPackageStartupMessages(library(lme4))

suppressPackageStartupMessages(library(lmerTest))

citation('lme4')$textVersion # so you have the  LMER version number

## [1] "Douglas Bates, Martin Maechler, Ben Bolker, Steve Walker (2015). Fitting Li
near Mixed-Effects Models Using lme4. Journal of Statistical Software, 67(1), 1-48.
doi:10.18637/jss.v067.i01."

#Design is 2 x 2 (group x type)

#group is between subjects (dyslexia vs. control)

#type is within subject (subject vs. object)


#all DVs are linear, except regressions which are binomial.
```

#Noun ##Total DV

```
noun<-read.csv("~/Desktop/paul_noun.csv")
```

```
noun$group<-as.factor(noun$group)

noun$type<-as.factor(noun$type)

noun$subject<-as.factor(noun$subject)

noun1<-noun[noun$total!=".",]#remove "." trials


noun_model<-lmer(total ~ group*type+(1+type|subject) + (1+group|item), data=noun1, 
REML=FALSE)

## boundary (singular) fit: see ?isSingular

## Warning: Model failed to converge with 1 negative eigenvalue: -2.1e+03

noun_modelre1<-lmer(total ~ group*type+(1+type|subject) + (1|item), data=noun1, REM
L=FALSE)

## boundary (singular) fit: see ?isSingular

noun_modelre2<-lmer(total ~ group*type+(1|subject) + (1|item), data=noun1, REML=FAL
SE)

summary(noun_modelre2)

## Linear mixed model fit by maximum likelihood . t-tests use Satterthwaite's

##   method [lmerModLmerTest]

## Formula: total ~ group * type + (1 | subject) + (1 | item)

##    Data: noun1

##

##      AIC      BIC   logLik deviance df.resid

##  31011.5  31050.7 -15498.8  30997.5     1993

##

## Scaled residuals:

##     Min      1Q  Median      3Q     Max

## -2.6555 -0.5696 -0.1497  0.3359 11.8619

##

## Random effects:

##  Groups   Name        Variance Std.Dev.

##  subject  (Intercept)  78786   280.7

##  item     (Intercept)  13086   114.4

##  Residual             282075   531.1

## Number of obs: 2000, groups:  subject, 100; item, 20

##

## Fixed effects:

##                      Estimate Std. Error      df t value Pr(>|t|)

## (Intercept)            656.83      52.87  116.47  12.424  < 2e-16 ***

## groupdyslexia          162.49      65.44  132.46   2.483   0.0143 *

## type2                 -133.99      33.63 1882.91  -3.984 7.05e-05 ***

## groupdyslexia:type2     76.91      47.63 1884.89   1.615   0.1065
```

```
## ---
## Signif. codes:  0 '***' 0.001 '**' 0.01 '*' 0.05 '.' 0.1 ' ' 1
##
## Correlation of Fixed Effects:
##             (Intr) grpdys type2
## groupdyslex -0.619
## type2       -0.318  0.258
## grpdyslx:t2  0.225 -0.364 -0.708
```

##First DV

```
noun2<-noun[noun$first!=".",] #remove "." trials
noun2$first<-as.numeric(as.character(noun2$first)) #convert to numeric
noun_model_first<-lmer(first ~ group*type+(1+type|subject) + (1+group|item), data=n
oun2, REML=FALSE)
## boundary (singular) fit: see ?isSingular
noun_model_first2<-lmer(first ~ group*type+(1+type|subject) + (1|item), data=noun2,
REML=FALSE)
## boundary (singular) fit: see ?isSingular
noun_model_first3<-lmer(first ~ group*type+(1|subject) + (1|item), data=noun2, REML
=FALSE)
summary(noun_model_first3)
## Linear mixed model fit by maximum likelihood . t-tests use Satterthwaite's
##   method [lmerModLmerTest]
## Formula: first ~ group * type + (1 | subject) + (1 | item)
##    Data: noun2
##
##      AIC      BIC   logLik deviance df.resid
##  24354.6  24393.4 -12170.3  24340.6     1872
##
## Scaled residuals:
##     Min      1Q  Median      3Q     Max
## -2.1979 -0.5940 -0.2246  0.3361 11.8082
##
## Random effects:
##  Groups   Name        Variance Std.Dev.
##  subject  (Intercept)  2411.2   49.10
##  item     (Intercept)   291.4   17.07
##  Residual             23166.6  152.21
## Number of obs: 1879, groups:  subject, 100; item, 20
##
```

```
## Fixed effects:
##                        Estimate Std. Error        df t value Pr(>|t|)
## (Intercept)            256.2049    10.6344  141.8904  24.092   <2e-16 ***
## groupdyslexia           -0.6669    14.0141  181.6654  -0.048   0.9621
## type2                   25.3999    10.0087 1772.7123   2.538   0.0112 *
## groupdyslexia:type2     19.5568    14.0880 1774.5566   1.388   0.1653
## ---
## Signif. codes:  0 '***' 0.001 '**' 0.01 '*' 0.05 '.' 0.1 ' ' 1
##
## Correlation of Fixed Effects:
##             (Intr) grpdys type2
## groupdyslex -0.661
## type2       -0.473  0.359
## grpdyslx:t2  0.336 -0.507 -0.711
```

##Regress DV

```
noun3<-noun[noun$regress!=".",]
str(noun3$regress)
##  chr [1:1879] "1" "0" "1" "0" "0" "0" "0" "0" "1" "0" "0" "0" "0" "0" "0" ...
noun3$regress<-as.numeric(as.character(noun3$regress))
noun_model_regress<-glmer(regress ~ group*type+(1+type|subject) + (1+group|item), d
ata=noun3,  family="binomial")
## boundary (singular) fit: see ?isSingular
noun_model_regress_re1<-glmer(regress ~ group*type+(1+type|subject) + (1|item), dat
a=noun3,  family="binomial")
summary(noun_model_regress_re1)
## Generalized linear mixed model fit by maximum likelihood (Laplace
##   Approximation) [glmerMod]
##  Family: binomial  ( logit )
## Formula: regress ~ group * type + (1 + type | subject) + (1 | item)
##    Data: noun3
##
##      AIC      BIC   logLik deviance df.resid
##   1982.5   2026.8   -983.3   1966.5     1871
##
## Scaled residuals:
##     Min      1Q  Median      3Q     Max
## -0.8004 -0.5492 -0.4712 -0.3724  2.7045
##
## Random effects:
```

```
##  Groups   Name        Variance Std.Dev. Corr
##  subject (Intercept) 0.191397 0.43749
##          type2       0.024986 0.15807  0.17
##  item    (Intercept) 0.002544 0.05044
## Number of obs: 1879, groups:  subject, 100; item, 20
##
## Fixed effects:
##                      Estimate Std. Error z value Pr(>|z|)
## (Intercept)          -1.24326    0.13147  -9.457   <2e-16 ***
## groupdyslexia         0.22301    0.17715   1.259   0.2081
## type2                -0.39755    0.17707  -2.245   0.0248 *
## groupdyslexia:type2  0.04047    0.23033   0.176   0.8605
## ---
## Signif. codes:  0 '***' 0.001 '**' 0.01 '*' 0.05 '.' 0.1 ' ' 1
##
## Correlation of Fixed Effects:
##             (Intr) grpdys type2
## groupdyslex -0.708
## type2       -0.558  0.392
## grpdyslx:t2  0.407 -0.566 -0.712
```

##regpath DV

```
noun4<-noun[noun$regpath!=".",]
str(noun4$regpath)
##  chr [1:1879] "1562" "165" "1768" "412" "163" "300" "1127" "704" "1355" ...
noun4$regpath<-as.numeric(as.character(noun4$regpath))


noun_mode_regpa<-lmer(regpath ~ group*type+(1+type|subject) + (1+group|item), data=
noun4, REML=FALSE)
## boundary (singular) fit: see ?isSingular
## Warning: Model failed to converge with 1 negative eigenvalue: -1.6e-01
noun_mode_regpa_re1<-lmer(regpath ~ group*type+(1+type|subject) + (1|item), data=no
un4, REML=FALSE)
## boundary (singular) fit: see ?isSingular
noun_mode_regpa_re2<-lmer(regpath ~ group*type+(1|subject) + (1|item), data=noun4,
REML=FALSE)
summary(noun_mode_regpa_re2)
## Linear mixed model fit by maximum likelihood . t-tests use Satterthwaite's
##   method [lmerModLmerTest]
## Formula: regpath ~ group * type + (1 | subject) + (1 | item)
```

```
##    Data: noun4
##
##      AIC      BIC   logLik deviance df.resid
##  29647.3  29686.0 -14816.6  29633.3     1872
##
## Scaled residuals:
##     Min      1Q  Median      3Q     Max
## -1.9890 -0.4764 -0.2689  0.0856 11.2639
##
## Random effects:
##  Groups   Name        Variance Std.Dev.
##  subject  (Intercept)  39874   199.69
##  item     (Intercept)   1478    38.44
##  Residual             389537   624.13
## Number of obs: 1879, groups:  subject, 100; item, 20
##
## Fixed effects:
##                      Estimate Std. Error      df t value Pr(>|t|)
## (Intercept)            473.23      41.43  156.91  11.423  < 2e-16 ***
## groupdyslexia          195.45      57.22  183.92   3.416 0.000783 ***
## type2                  -26.99      41.02 1775.47  -0.658 0.510709
## groupdyslexia:type2    -47.66      57.72 1778.63  -0.826 0.409069
## ---
## Signif. codes:  0 '***' 0.001 '**' 0.01 '*' 0.05 '.' 0.1 ' ' 1
##
## Correlation of Fixed Effects:
##             (Intr) grpdys type2
## groupdyslex -0.693
## type2       -0.497  0.360
## grpdyslx:t2  0.354 -0.509 -0.711
```

#Verb

```
verb<-read.csv("~/Desktop/paul_verb.csv")
verb$group<-as.factor(verb$group)
verb$type<-as.factor(verb$type)
verb$subject<-as.factor(verb$subject)
```

##Total DV

```
verb1<-verb[verb$total!=".",]#remove "." trials
```

```
verb_model<-lmer(total ~ group*type+(1+type|subject) + (1+group|item), data=verb1,
REML=FALSE)
## boundary (singular) fit: see ?isSingular
verb_modelre1<-lmer(total ~ group*type+(1+type|subject) + (1|item), data=verb1, REM
L=FALSE)


summary(verb_modelre1)
## Linear mixed model fit by maximum likelihood . t-tests use Satterthwaite's
##   method [lmerModLmerTest]
## Formula: total ~ group * type + (1 + type | subject) + (1 | item)
##    Data: verb1
##
##      AIC      BIC   logLik deviance df.resid
##  31819.3  31869.7 -15900.6  31801.3     1991
##
## Scaled residuals:
##     Min      1Q  Median      3Q     Max
## -2.6227 -0.5658 -0.1549  0.3738 11.3391
##
## Random effects:
##  Groups   Name        Variance Std.Dev. Corr
##  subject  (Intercept) 121869   349.1
##           type2        19534   139.8    -0.43
##  item     (Intercept)  32215   179.5
##  Residual             417345   646.0
## Number of obs: 2000, groups:  subject, 100; item, 20
##
## Fixed effects:
##                      Estimate Std. Error      df t value Pr(>|t|)
## (Intercept)            867.25      69.89   86.61  12.409  < 2e-16 ***
## groupdyslexia          267.61      80.93   99.91   3.307 0.001312 **
## type2                 -163.49      45.45   99.35  -3.597 0.000504 ***
## groupdyslexia:type2     44.26      64.36   99.43   0.688 0.493277
## ---
## Signif. codes:  0 '***' 0.001 '**' 0.01 '*' 0.05 '.' 0.1 ' ' 1
##
## Correlation of Fixed Effects:
##             (Intr) grpdys type2
## groupdyslex -0.579
```

```
## type2        -0.396  0.343
## grpdyslx:t2  0.280 -0.484 -0.708
```

##First DV

```
verb2<-verb[verb$first!=".",] #remove "." trials

verb2$first<-as.numeric(as.character(verb2$first)) #convert to numeric

verb_model_first<-lmer(first ~ group*type+(1+type|subject) + (1+group|item), data=v
erb2, REML=FALSE)

## boundary (singular) fit: see ?isSingular

verb_model_first2<-lmer(first ~ group*type+(1+type|subject) + (1|item), data=verb2,
REML=FALSE)


summary(verb_model_first2)

## Linear mixed model fit by maximum likelihood . t-tests use Satterthwaite's
##   method [lmerModLmerTest]
## Formula: first ~ group * type + (1 + type | subject) + (1 | item)
##    Data: verb2
##
##      AIC      BIC   logLik deviance df.resid
##  26239.8  26290.0 -13110.9  26221.8     1938
##
## Scaled residuals:
##     Min      1Q  Median      3Q     Max
## -2.1989 -0.6087 -0.2334  0.3874  6.6555
##
## Random effects:
##  Groups   Name        Variance Std.Dev. Corr
##  subject  (Intercept)  4700     68.56
##           type2        1689     41.10   -0.70
##  item     (Intercept)  1295     35.98
##  Residual             38479    196.16
## Number of obs: 1947, groups:  subject, 100; item, 20
##
## Fixed effects:
##                     Estimate Std. Error     df t value Pr(>|t|)
## (Intercept)           320.31      15.43  86.53  20.763   <2e-16 ***
## groupdyslexia          54.46      18.59 100.40   2.930   0.0042 **
## type2                 -27.85      13.90 100.66  -2.003   0.0478 *
## groupdyslexia:type2   -20.87      19.63  99.94  -1.063   0.2905
## ---
```

```
## Signif. codes:  0 '***' 0.001 '**' 0.01 '*' 0.05 '.' 0.1 ' ' 1
##
## Correlation of Fixed Effects:
##             (Intr) grpdys type2
## groupdyslex -0.605
## type2       -0.555  0.461
## grpdyslx:t2  0.393 -0.649 -0.709
```

##Regress DV

```
verb3<-verb[verb$regress!=".",]
str(verb3$regress)
##  chr [1:1947] "0" "1" "0" "0" "0" "0" "0" "0" "0" "0" "1" "1" "0" "0" "0" ...
verb3$regress<-as.numeric(as.character(verb3$regress))
verb_model_regress<-glmer(regress ~ group*type+(1+type|subject) + (1+group|item), d
ata=verb3,  family="binomial")
## boundary (singular) fit: see ?isSingular
verb_model_regress_re1<-glmer(regress ~ group*type+(1+type|subject) + (1|item), dat
a=verb3,  family="binomial")
summary(verb_model_regress_re1)
## Generalized linear mixed model fit by maximum likelihood (Laplace
##   Approximation) [glmerMod]
##  Family: binomial  ( logit )
## Formula: regress ~ group * type + (1 + type | subject) + (1 | item)
##    Data: verb3
##
##      AIC      BIC   logLik deviance df.resid
##   2277.0   2321.6  -1130.5   2261.0     1939
##
## Scaled residuals:
##     Min      1Q  Median      3Q     Max
## -0.8924 -0.6272 -0.5347  1.2634  2.1984
##
## Random effects:
##  Groups  Name        Variance Std.Dev. Corr
##  subject (Intercept) 0.261410 0.51128
##          type2       0.009979 0.09989  -1.00
##  item    (Intercept) 0.006755 0.08219
## Number of obs: 1947, groups:  subject, 100; item, 20
##
## Fixed effects:
```

```
##                        Estimate Std. Error z value Pr(>|z|)
## (Intercept)           -1.20674    0.13524  -8.923   <2e-16 ***
## groupdyslexia          0.19755    0.18159   1.088    0.277
## type2                  0.09746    0.15653   0.623    0.534
## groupdyslexia:type2    0.12066    0.20924   0.577    0.564
## ---
## Signif. codes:  0 '***' 0.001 '**' 0.01 '*' 0.05 '.' 0.1 ' ' 1
##
## Correlation of Fixed Effects:
##              (Intr) grpdys type2
## groupdyslex -0.707
## type2       -0.645  0.459
## grpdyslx:t2  0.461 -0.642 -0.720
```

##regpath DV

```
verb4<-verb[verb$regpath!=".",]
str(verb4$regpath)
##  chr [1:1947] "169" "1122" "217" "189" "221" "169" "356" "146" "386" "146" ...
verb4$regpath<-as.numeric(as.character(verb4$regpath))


verb_mode_regpa<-lmer(regpath ~ group*type+(1+type|subject) + (1+group|item), data=
verb4, REML=FALSE)
## boundary (singular) fit: see ?isSingular
verb_mode_regpa_re1<-lmer(regpath ~ group*type+(1+type|subject) + (1|item), data=ve
rb4, REML=FALSE)


summary(verb_mode_regpa_re1)
## Linear mixed model fit by maximum likelihood . t-tests use Satterthwaite's
##   method [lmerModLmerTest]
## Formula: regpath ~ group * type + (1 + type | subject) + (1 | item)
##    Data: verb4
##
##      AIC      BIC   logLik deviance df.resid
##  30556.6  30606.7 -15269.3  30538.6     1938
##
## Scaled residuals:
##     Min      1Q  Median      3Q     Max
## -2.1504 -0.5511 -0.2788  0.2671  7.4969
##
## Random effects:
```

```
##  Groups   Name        Variance Std.Dev. Corr
##  subject  (Intercept)  68919   262.52
##           type2        13897   117.88   -0.83
##  item     (Intercept)   6482    80.51
##  Residual             349776   591.42
## Number of obs: 1947, groups:  subject, 100; item, 20
##
## Fixed effects:
##                      Estimate Std. Error     df t value Pr(>|t|)
## (Intercept)            598.05      49.22 103.22  12.150   <2e-16 ***
## groupdyslexia          164.22      64.72 100.38   2.538   0.0127 *
## type2                  -91.98      41.55 102.21  -2.214   0.0291 *
## groupdyslexia:type2     24.84      58.68 101.50   0.423   0.6729
## ---
## Signif. codes:  0 '***' 0.001 '**' 0.01 '*' 0.05 '.' 0.1 ' ' 1
##
## Correlation of Fixed Effects:
##             (Intr) grpdys type2
## groupdyslex -0.659
## type2       -0.602  0.459
## grpdyslx:t2  0.427 -0.646 -0.709
```

Section C: Item Analyses

Table A

Mixed ANCOVA item analysis for eye movement measures for the relative verb

	**First Pass RT**	**Total RT**	**Regressions Out**	**Regression Path**
**2 x 2 (Type x Group)**				
Type	$F(1,19) = 13.67, p < .01$	$F(1,19) = 12.82, p < .01$	N.S.	$F(1,19) = 5.24, p < .05$
Group	$F(1,19) = 16.52, p < .01$	$F(1,19) = 48.97, p < .001$	$F(1,19) = 7.81, p < .05$	$F(1,19) = 50.03, p < .001$
Type x Group	N.S.	N.S.	N.S.	N.S.
**ANCOVA – with Verbal IQ**				
Type	$F(1,18) = 14.68, p < .01$	$F(1,18) = 25.96, p < .001$	N.S.	$F(1,18) = 7.36, p < .05$
Group	$F(1,18) = 11.90, p < .01$	$F(1,18) = 37.22, p < .001$	$F(1,18) = 6.05, p < .05$	$F(1,18) = 39.02, p < .001$
Verbal IQ	N.S.	N.S.	N.S.	N.S.
Type x Group	N.S.	N.S.	N.S.	N.S.
Type x Verbal IQ	N.S.	$F(1,18) = 8.91, p < .01$	N.S.	N.S.
**ANCOVA – with WM**				
Type	N.S.	$F(1,18) = 5.17, p < .05$	N.S.	N.S.
Group	N.S.	$F(1,18) = 6.49, p < .05$	N.S.	N.S.
Working Memory	N.S.	N.S.	N.S.	N.S.
Type x Group	N.S.	N.S.	N.S.	N.S.
Type x Working Memory	N.S.	N.S.	N.S.	N.S.

Table B

Mixed ANCOVA item analysis for eye movement measures for the relative noun

	**First Pass RT**	**Total RT**	**Regressions Out**	**Regression Path**
**2 x 2 (Type x Group)**				
Type	$F(1,19) = 21.44$, $p < .001$	$F(1,19) = 17.31$, $p < .01$	$F(1,19) = 9.16$, $p < .01$	N.S.
Group	N.S.	$F(1,19) = 39.50$, $p < .001$	$F(1,19) = 5.37$, $p < .05$	$F(1,19) = 27.28$, $p < .001$
Type x Group	N.S.	N.S.	N.S.	N.S.
**ANCOVA – with Verbal IQ**				
Type	$F(1,18) = 19.22$, $p < .001$	$F(1,18) = 18.02$, $p < .001$	$F(1,18) = 5.39$, $p < .05$	N.S.
Group	N.S.	$F(1,18) = 33.11$, $p < .001$	N.S.	$F(1,18) = 19.56$, $p < .001$
Verbal IQ	N.S.	N.S.	N.S.	N.S.
Type x Group	N.S.	N.S.	N.S.	N.S.
Type x Verbal IQ	N.S.	N.S.	N.S.	N.S.
**ANCOVA – with WM**				
Type	$F(1,18) = 5.21$, $p < .05$	$F(1,18) = 7.23$, $p < .05$	N.S.	N.S.
Group	N.S.	$F(1,18) = 8.03$, $p < .05$	N.S.	N.S.
Working Memory	N.S.	N.S.	N.S.	N.S.
Type x Group	N.S.	N.S.	N.S.	N.S.
Type x Working Memory	N.S.	N.S.	N.S.	N.S.
